# Retrograde Transgene Expression via Neuron-Specific Lentiviral Vector Depends on Both Species and Input Projections

**DOI:** 10.3390/v13071387

**Published:** 2021-07-16

**Authors:** Yukiko Otsuka, Hitomi Tsuge, Shiori Uezono, Soshi Tanabe, Maki Fujiwara, Miki Miwa, Shigeki Kato, Katsuki Nakamura, Kazuto Kobayashi, Ken-ichi Inoue, Masahiko Takada

**Affiliations:** 1 Systems Neuroscience Section, Department of Neuroscience, Primate Research Institute, Kyoto University, Inuyama 484-8506, Aichi, Japan; otsuka.yukiko.37s@st.kyoto-u.ac.jp (Y.O.); soraironokumo@gmail.com (H.T.); s-uezono@u-ths.ac.jp (S.U.); tanabe.soshi.67v@gmail.com (S.T.); fujiwara.maki.2x@kyoto-u.ac.jp (M.F.); 2 Department of Rehabilitation, Faculty of Health Science, University of Tokyo Health Sciences, Tama, Tokyo 206-0033, Japan; 3 Cognitive Neuroscience Section, Department of Neuroscience, Primate Research Institute, Kyoto University, Inuyama 484-8506, Aichi, Japan; miwa.miki.8m@kyoto-u.ac.jp (M.M.); nakamura.katsuki.4z@kyoto-u.ac.jp (K.N.); 4 Department of Molecular Genetics, Institute of Biomedical Sciences, Fukushima Medical University School of Medicine, Fukushima 960-1295, Fukushima, Japan; skato@fmu.ac.jp (S.K.); kazuto@fmu.ac.jp (K.K.)

**Keywords:** lentiviral vector, pseudotyping, retrograde gene transfer, cerebral cortex, thalamus, neuron specificity, inflammation, primates

## Abstract

For achieving retrograde gene transfer, we have so far developed two types of lentiviral vectors pseudotyped with fusion envelope glycoprotein, termed HiRet vector and NeuRet vector, consisting of distinct combinations of rabies virus and vesicular stomatitis virus glycoproteins. In the present study, we compared the patterns of retrograde transgene expression for the HiRet vs. NeuRet vectors by testing the cortical input system. These vectors were injected into the motor cortex in rats, marmosets, and macaques, and the distributions of retrograde labels were investigated in the cortex and thalamus. Our histological analysis revealed that the NeuRet vector generally exhibits a higher efficiency of retrograde gene transfer than the HiRet vector, though its capacity of retrograde transgene expression in the macaque brain is unexpectedly low, especially in terms of the intracortical connections, as compared to the rat and marmoset brains. It was also demonstrated that the NeuRet but not the HiRet vector displays sufficiently high neuron specificity and causes no marked inflammatory/immune responses at the vector injection sites in the primate (marmoset and macaque) brains. The present results indicate that the retrograde transgene efficiency of the NeuRet vector varies depending not only on the species but also on the input projections.

## 1. Introduction

Lentiviral vectors based on HIV (human immunodeficiency virus)-1 have been developed for gene transfer experiments in the brain [1,2,3]. In general, a viral vector that allows gene transfer through retrograde axonal transport infects axon terminals of neurons at its injection site and expresses the transgene at cell bodies in a region projecting to the injection site. Since such a retrograde infection-type vector provides a powerful tool for pathway-selective manipulation and monitoring of neuronal activity, recent attention has been paid to pseudotyped lentiviral vectors [4,5,6,7,8,9,10,11,12,13]. To confer retrograde infection capacity on ordinary lentiviral vectors, an attempt was initially made at pseudotyping with rabies virus glycoprotein (RV-G) as a substitute for vesicular stomatitis virus glycoprotein (VSV-G) [5,6]. After several trials, we have so far created two types of lentiviral vectors pseudotyped with fusion envelope glycoprotein (FuG), termed HiRet vector and NeuRet vector, which consist of distinct combinations of RV-G and VSV-G [9,10,11,12,13]. The HiRet vector is pseudotyped with FuG-B2, consisting of the extracellular and transmembrane domain of RV-G and the membrane proximal regions of VSV-G, while the NeuRet vector is pseudotyped with FuG-E, consisting of the N-terminal segment of RV-G and the membrane proximal region and the transmembrane/cytoplasmic domains of VSV-G. Both of these vectors successfully achieve highly efficient retrograde gene transfer.

To further compare the properties of the HiRet (FuG-B2 type) vs. NeuRet (FuG-E type) vectors in terms of neuron specificity and inflammatory events as well as retrograde transgene efficiency, we previously analyzed the differential patterns of transgene expression within the cells of origin of striatal inputs in macaques, marmosets, and rats [14]. It was revealed in our work that the NeuRet vector generally possesses superiority to the HiRet vector across the three species in all the considerations listed above. Moreover, the retrograde transgene efficacy for the NeuRet vector has been found to vary depending not only on the species but also on the input source (i.e., whether the input pathway arises from the cerebral cortex, thalamus, or substantia nigra).

In the present study, we tested the cortical input system, especially directed toward the motor cortex, to compare the patterns of retrograde transgene expression for the HiRet and NeuRet vectors among the same three species. Recently, we further constructed FuG-E variants with single amino acid substitutions at position 440 in the membrane-proximal region of FuG-E and found that the substitution of proline at residue 440 for glutamate represents the highest retrograde gene transfer efficiency [15]. Together with the FuG-B2 type of HiRet vector (Addgene plasmid ID number 67513) and the FuG-E type of NeuRet vector (Addgene plasmid ID number 67509), the FuG-E (P440E) variant of NeuRet vector (Addgene plasmid ID number 119978) was used for the present purpose. Here we show that the NeuRet vector is, on the whole, more appropriate than the HiRet vector for retrograde gene delivery into the cortical input system, although its retrograde transgene efficacy in the macaque brain is unexpectedly low as compared to the marmoset and rat brains.

## 2. Methods

### 2.1. Animals

Twelve male Wistar rats (250–350 g), six common marmosets of either sex (250–360 g), and four rhesus macaques of either sex (4.6–6.2 kg) were used for this study. The experimental procedure was approved by the Animal Welfare and Animal Care Committee of the Primate Research Institute, Kyoto University (Permission Number: 2017-031), and all experiments were conducted according to the Guidelines for Care and Use of Nonhuman Primates established by the Primate Research Institute, Kyoto University (2010). Since the Guidelines required us to consider 3R-type recommendations in designing monkey experiments, we decided to use one or two animals for each purpose. All experiments were performed in a special laboratory (biosafety level 2) that had been established at the Primate Research Institute, Kyoto University, for in vivo animal infectious experiments. During the experiments, the animals were housed in individual cages, which were placed inside a special safety cabinet with controlled temperature (23–26 °C for rats and macaques; 26–30 °C for marmosets) and light (12 h on/off cycle) conditions. The animals were fed regularly with dietary pellets and had ad libitum access to water. Every effort was made to minimize animal suffering.

### 2.2. Viral Vector Production

Construction of envelope plasmids (pCAGGS-FuG-B2, pCAGGS-FuG-E, and pCAGGS-FuG-E (P400E)) and recovery of viral vectors were performed as described previously [10,12,15]. In short, HEK293T cells were transfected with transfer (pCL20c-MSCV-EGFP), envelope, and packaging (pCAG-kGP4.1R and pCAG4-RTR2) plasmids by the calcium-phosphate precipitation method. Eighteen hours after transfection, the medium was replaced with a fresh one, and thereafter, cells were incubated for 24 h. Then, the medium was harvested and filtered through a 0.45 µm Millex-HV filter unit (Millipore, Burlington, MA, USA). Viral vector particles were pelleted by centrifugation at 6000× *g* for 16–18 h and resuspended in 0.01 M phosphate-buffered saline (PBS; pH 7.4). The particles were then applied to a Sepharose Q FF ion-exchange column (GE Healthcare, Buckinghamshire, UK) in 0.01 M PBS and eluted with a linear 0–1.5 M NaCl gradient. The fractions were monitored at 260/280 nm of absorbance wavelength. The peak fractions containing the particles were collected and concentrated to 110 µL by centrifugation through a Vivaspin filter (Vivascience, Lincoln, UK).

For measuring RNA titer, viral RNA in 50 nL of the vector stock solution was isolated with a NucleoSpin RNA virus kit (Takara, Shiga, Japan), and the copy number of the RNA genome was determined by quantitative PCR using Taq-Man technology (Thermo Fisher Scientific, Waltham, MA, USA). For each vector, the same RNA titer rather than functional titer was used [14].

### 2.3. Surgical Procedures

The rats were first sedated with isoflurane (1–5%, inhalation) and then anesthetized with ketamine hydrochloride (50 mg/kg, i.m.) and xylazine hydrochloride (4 mg/kg, i.m.). A volume of 0.5 µL of each vector was injected at the titer of 1.0 × 10^10^ gc/mL into the secondary motor cortex (M2) through a glass microinjection capillary connected to a microinfusion pump. For the M2, the coordinates from the bregma and dura were 4.0/1.5/1.0 mm according to an atlas of the rat brain [16].

The marmosets were first sedated with ketamine hydrochloride (50 mg/kg, i.m.) and xylazine hydrochloride (2 mg/kg, i.m.) and then anesthetized with sodium pentobarbital (15 mg/kg, i.p.). An antibiotic (Cefmetazole; 25 mg/kg, i.m.) and 10 mL of acetated Ringer’s solution (s.c.) were administered before and after the operation. After partial removal of the skull, multiple injections of each vector were performed into Brodmann’s area 6m (A6m), measured by magnetic resonance imaging (MRI). A total volume of 2 µL of each vector was injected at the titer of 1.0 × 10^10^ gc/mL into four sites (0.5 µL/site) through a 10 µL Hamilton microsyringe. After the injections were complete, the scalp incision was closed, and an analgesic (Meloxicam; 1 mg/kg, s.c.) was administered.

The macaque monkeys were first sedated with ketamine hydrochloride (5 mg/kg, i.m.) and xylazine hydrochloride (0.5 mg/kg, i.m.) and then anesthetized with sodium pentobarbital (20 mg/kg, i.v.). During the surgical operation, the monkeys were kept hydrated with a lactated Ringer’s solution (i.v.). An antibiotic (Ceftazidime; 25 mg/kg, i.v.) and an analgesic (Meloxicam; 0.2 mg/kg, s.c.) were administered at the first anesthesia. After partial removal of the skull, multiple injections of each vector were performed into the supplementary motor area (SMA) by the aid of an MRI-guided navigation system (Brainsight Primate, Rogue Research, Montreal, QC, Canada). A total volume of 4.5 µL of each vector was injected into multiple sites (0.5 µL/site, three sites per track, three to five tracks per monkey) through a 10 µL Hamilton microsyringe. The injection titer of the HiRet vector was 8.5 × 10^9^ gc/mL, that of the NeuRet (FuG-E) vector was 8.5 × 10^9^ gc/mL or 4.0 × 10^10^ gc/mL, and that of the NeuRet (P440E) vector was 4.0 × 10^10^ gc/mL. After the injections were complete, the scalp incision was closed.

### 2.4. Immunohistochemistry

Four weeks after the vector injections, the animals were anesthetized deeply with an overdose of sodium pentobarbital (100 mg/kg, i.p. for rats; 50 mg/kg, i.p. for marmosets; 50 mg/kg, i.v. for macaques) and perfused transcardially with 0.1 M PBS, followed by 10% formalin in 0.1 M phosphate buffer. The fixed brains were removed from the skull, postfixed in the same fresh fixative overnight at 4 °C, and equilibrated with 30% sucrose in 0.1 M PBS at 4 °C. Coronal sections were cut serially on a freezing microtome at a 40 µm thickness for rats and marmosets and a 50 µm thickness for macaques. Every tenth section was mounted onto gelatin-coated glass slides and Nissl-stained with 1% Cresyl violet.

For immunoperoxidase staining, the sections were pretreated with 0.3% H_2_O_2_ for 30 min, washed three times in 0.1 M PBS, and immersed in 1% skim milk for 1 h. Subsequently, the sections were incubated for two days at 4 °C with rabbit polyclonal anti-green fluorescent protein (GFP) antibody (1:2000 dilution; Invitrogen, Waltham, MA, USA) in 0.1 M PBS containing 2% normal donkey serum and 0.1% Triton X-100. The sections were incubated with biotinylated donkey anti-rabbit IgG antibody (1:1000 dilution; Jackson Immuno Research Laboratories, West Grove, PA, USA) in the same fresh medium for 2 h at room temperature, then incubated with the avidin-biotin-peroxidase complex kit (ABC Elite; 1:200 dilution; Vector Laboratories, Burlingame, CA, USA) in 0.1 M PBS for 1.5 h at room temperature. To visualize the antigen, the sections were reacted for 10–20 min in 0.05 M Tris-HCl buffer (pH 7.6) containing 0.04% diaminobenzidine tetrahydrochloride (Wako, Osaka, Japan), 0.04% NiCl_2_, and 0.002% H_2_O_2_. The reaction time was adjusted to the cases. These sections were mounted onto gelatin-coated glass slides and counterstained with 0.5% Neutral red.

For double immunofluorescence histochemistry for ionized calcium-binding adapter molecule 1 (Iba1) and cluster of differentiation 8 (CD8), the sections were pretreated with 0.3% H_2_O_2_ for 30 min, washed three times in 0.1 M PBS, and immersed with 0.5% Blocking Reagent (NEL703001KT; PerkinElmer, Waltham, MA, USA) in 0.1 M PBS for 1 h. The sections were then incubated for two days at 4 °C with rabbit monoclonal anti-Iba1 antibody (1:1000 dilution; Wako) and mouse monoclonal anti-rat CD8 antibody (1:1000 dilution; Bio-Rad, Berkeley, CA, USA), anti-marmoset CD8 antibody (1:4000 dilution; BioLegend, San Diego, CA, USA), or anti-human CD8 antibody (1:2000 dilution; Bio-Rad) in 0.1 M PBS containing 1% normal donkey serum and 0.1% Triton X-100. The sections were incubated with peroxidase-conjugated donkey anti-mouse IgG antibody (1:250 dilution; Jackson Immuno Research Laboratories) in the same fresh medium for 2 h at room temperature, then immersed with Biotin Plus Amplification Reagent in 1xPlus Amplification Diluent (1:50 dilution; TSA Plus Biotin Kit; PerkinElmer) for 10 min at room temperature. Subsequently, the sections were incubated for 2 h at room temperature with a cocktail of DyLight 405-conjugated streptavidin (1:100 dilution; Jackson Immuno Research Laboratories) and Alexa 647-conjugated donkey ant-rabbit IgG antibody (1:200 dilution; Jackson Immuno Research Laboratories) in 0.1 M PBS containing 1% normal donkey serum.

For double immunofluorescence histochemistry for GFP and NeuN or GFP and glial fibrillary acid protein (GFAP), the sections were rinsed three times in 0.1 M PBS, immersed in 1% skim milk for 1 h, and then incubated for two days with sheep polyclonal anti-GFP antibody (1:20,000 dilution; Thermo Fisher Scientific) and mouse monoclonal antibodies as well as anti-NeuN antibody (1:2000 dilution; Millipore) or anti-GFAP antibody (1:500 dilution; Sigma, St. Louis, MO, USA). The sections were then incubated for 2 h at room temperature with a cocktail of Alexa 488-conjugated donkey anti-sheep IgG antibody (1:400 dilution; Jackson Immuno Research Laboratories) and Alexa 647-conjugated donkey anti-mouse IgG antibody (1:400 dilution; Jackson Immuno Research Laboratories) in the same fresh medium.

### 2.5. Image Acquisition and Histological Analyses

Brightfield microscopic images were captured using an optical microscope equipped with a high-grade charge-coupled device (CCD) camera (Biorevo, Keyence, Osaka, Japan) or a scientific CMOS camera (In Cell Analyzer 2200, GE Healthcare). Fluorescent microscopic images were taken under a confocal laser-scanning microscope (LSM800, Carl Zeiss, White Plains, NY, USA). The number or density of GFP-positive neurons in each brain region was calculated with Neurolucida software (MicroBrightField, Williston, VT, USA) and Matlab software (Mathworks, Natick, MA, USA). For measurements of the density of GFP-positive neurons in the cortex, five to six equidistant sections were selected for each case. The total number of GFP-positive neurons and the total area examined were calculated for each cortical area. The number of GFP-positive neurons was divided by the total area to obtain the density of GFP-positive neurons. For counts of GFP-positive neurons in the thalamus, five equidistant sections were used in each case. For calculating the ratio of cells double-labeled for both GFP and NeuN or both GFP and GFAP to the total GFP-positive cells, five equidistant sections were used in each case.

## 3. Results

### 3.1. Injections of HiRet and NeuRet Vectors into Motor Cortex

For analyzing the efficiency of retrograde gene transfer into the striatal input system, we have so far produced the HiRet (FuG-B2) and NeuRet (FuG-E and P440E) vectors carrying the GFP gene [13,14]. In the present series of experiments, the same three vectors were utilized for investigating the patterns of retrograde transgene expression in the cortical input system. We injected these vectors into the medial part of the frontal motor cortex, corresponding to the M2 in rats, A6m in marmosets, and SMA in macaques. The injection titer of the vectors was set at the titer of 1.0 × 10^10^ gc/mL in the rat brain, 1.0 × 10^10^ gc/mL in the marmoset brain, and 8.5 × 10^9^ gc/mL in the macaque brain. For the macaque brain, the original NeuRet vector (FuG-E) was injected at a higher titer (4.0 × 10^10^ gc/mL) as well, because the production efficiency of this vector was higher than that of the HiRet vector [14]. Following intracortical injections of the HiRet (FuG-B2) and NeuRet (FuG-E and P440E) vectors, we examined the efficacy of retrograde gene delivery into the cortical input system for the HiRet vs. NeuRet vectors by comparing the patterns of retrograde transgene expression in the cortex and thalamus among the three species. As target cortical and thalamic regions, we selected the contralateral counterpart in the cortex, some ipsilateral cortical areas (i.e., the posterior parietal cortex (PE), the posterior cingulate cortex (PCC), and the ventral premotor cortex), and the motor thalamus, including the ventrolateral (VL) and centrolateral (CL) nuclei, where numbers of neurons are known to project to the medial frontal motor cortex [17,18,19].

### 3.2. Retrograde Transgene Expression via HiRet and NeuRet Vectors in Rats

**Figure 1:**
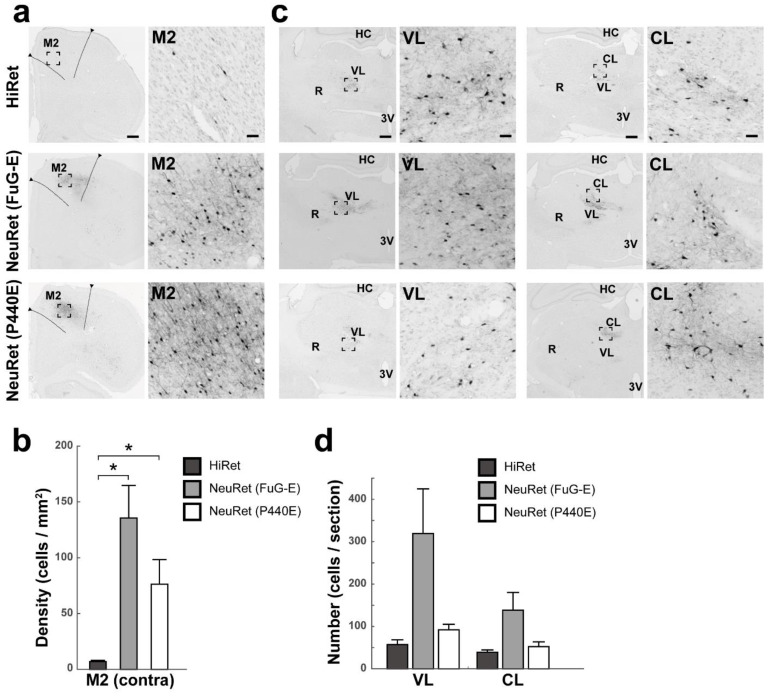
Retrograde transgene expression via HiRet and NeuRet vectors after vector injections into M2 in rats. (**a**) GFP immunostaining in the cortex (contralateral M2). Right panels denote higher-power magnifications of the square areas in left panels. Scale bars, 500 μm (left) and 50 μm (right). (**b**) Density (cells per mm^2^) of GFP-positive cells in the contralateral M2. Mean ± SEM (*n* = 4). * *p* < 0.05, significant differences from the value for the HiRet vector (Student’s *t* test). (**c**) GFP immunostaining in the ipsilateral thalamus (left, VL; right, CL). Right panels denote higher-power magnifications of the square areas in left panels. HC, hippocampus; R, reticular nucleus of the thalamus; 3V, third ventricle. Scale bars, 500 μm (left) and 50 μm (right). (**d**) Number (cells per section) of GFP-positive cells in the ipsilateral VL and CL. Data are expressed as the mean ± SEM (*n* = 4).

After the vector injections into the M2 of rats, the density of GFP-positive cortical neurons was evaluated in the contralateral M2 (Figure 1a). In the case of the HiRet, NeuRet (FuG-E), and NeuRet (P440E) vector injection, the mean density was 6.9 ± 1.2, 135.6 ± 29.2, and 76.3 ± 22.0 cells per mm^2^ (*n* = 4), respectively (Student’s *t* test, *p* < 0.05; Figure 1b). Counts of GFP-positive thalamic neurons were performed in the VL and CL on the ipsilateral side (Figure 1c). For the HiRet, NeuRet (FuG-E), and NeuRet (P440E) vector, the mean number in the VL was 57.3 ± 11.2, 319.3 ± 105.3, and 92 ± 13.1 cells per section (*n* = 4) and that in the CL was 39.0 ± 5.7, 138.5 ± 41.8, and 52.3 ± 11.5 cells per section (*n* = 4), respectively (Figure 1d). The overall data indicated that throughout the cortical and thalamic regions tested, much larger numbers of GFP-positive neurons were located after the injection of the NeuRet (FuG-E) vector than of the other vectors.

### 3.3. Retrograde Transgene Expression via HiRet and NeuRet Vectors in Marmosets

**Figure 2:**
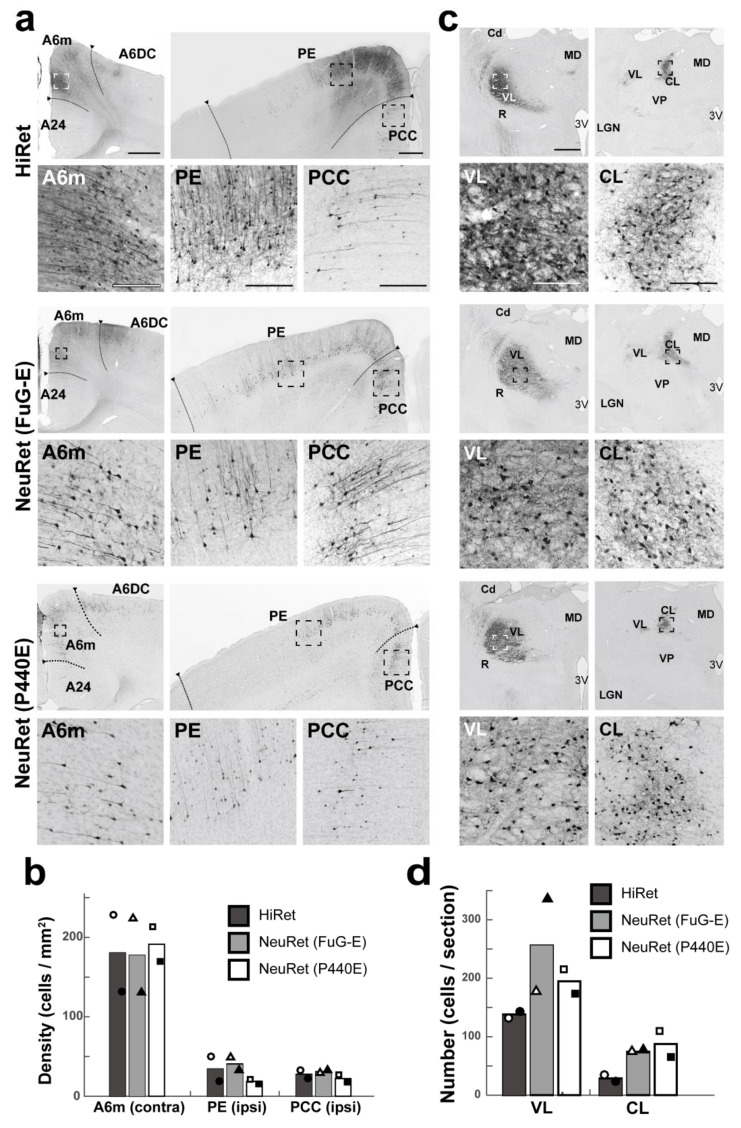
Retrograde transgene expression via HiRet and NeuRet vectors after vector injections into A6m in marmosets. (**a**) GFP immunostaining in the cortex (left, contralateral A6m; right, ipsilateral PE and PCC). A6DC, dorsocaudal part of area 6; A24, area 24. Scale bars, 500 µm (left) and 100 μm (right). (**b**) Density (cells per mm^2^) of GFP-positive cells in contralateral A6m and the ipsilateral PE and PCC. Data obtained in two animals are indicated with hollow and solid symbols (circles, triangles, and squares). (**c**) GFP immunostaining in the ipsilateral thalamus (left, VL; right, CL). Cd, caudate nucleus; LGN, lateral geniculate nucleus of the thalamus; MD, mediodorsal nucleus of the thalamus; VP, ventroposterior nucleus of the thalamus. Other abbreviations are as in Figure 1. Scale bars, 500 µm (left) and 100 μm (right). (**d**) Number (cells per section) of GFP-positive cells in the ipsilateral VL and CL. Data obtained in two animals are indicated with hollow and solid symbols (circles, triangles, and squares). All other conventions are as in Figure 1.

After the vector injections into A6m of marmosets, the distribution pattern of GFP-positive cortical neurons was examined in A6m contralaterally and, additionally, in the PE and PCC of the ipsilateral hemisphere (Figure 2a). The mean density of GFP-positive neurons in the contralateral A6m was high and comparable across the vectors: 180.5, 177.5, and 191.3 cells per mm^2^ (*n* = 2) for the HiRet, NeuRet (FuG-E), and NeuRet (P440E) vector, respectively. The mean density values in the ipsilateral PE and PCC were also comparable across the vectors, though relatively low compared to that in the contralateral A6m: for the PE, 34.4, 40.7, and 18.8 cells per mm^2^ (*n* = 2) and for the PCC, 27.7, 31.0, and 22.2 cells per mm^2^ (*n* = 2), respectively, in the case of the HiRet, NeuRet (FuG-E), and NeuRet (P440E) vector injection (Figure 2b). Many GFP-positive thalamic neurons were observed in the VL and CL on the side ipsilateral to the vector injections (Figure 2c). For the HiRet, NeuRet (FuG-E), and NeuRet (P440E) vector, the mean number in the VL was 138.1, 256.1, and 194.9 cells per section (*n* = 2) and that in the CL was 28.7, 74.2, and 87.5 cells per section (*n* = 2), respectively (Figure 2d). The overall data indicated that almost no differences were seen in the number of GFP-positive cortical neurons among the three vectors, while greater numbers of GFP-positive thalamic neurons were located after the injections of the NeuRet (FuG-E and P440E) vectors than of the HiRet vector.

### 3.4. Retrograde Transgene Expression via HiRet and NeuRet Vectors in Macaques

**Figure 3:**
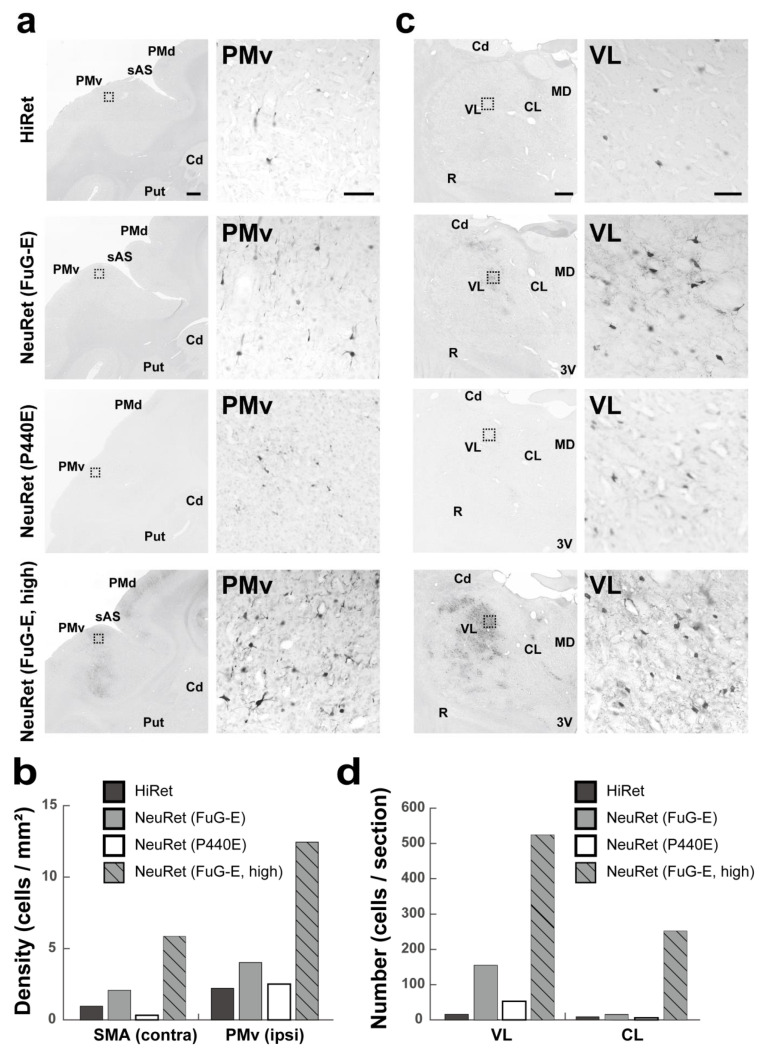
Retrograde transgene expression via HiRet and NeuRet vectors after vector injections into SMA in macaques. (**a**) GFP immunostaining in the cortex (ipsilateral ventral premotor cortex, PMv). Cd, caudate nucleus; PMd, dorsal premotor cortex; Put, putamen; sAS, spur of the arcuate sulcus. Scale bars, 1 mm (left) and 100 μm (right). (**b**) Density (cells per mm^2^) of GFP-positive cells in the contralateral SMA and the ipsilateral PMv. (**c**) GFP immunostaining in the ipsilateral thalamus (VL). All abbreviations are as in Figure 1 and Figure 2. (**d**) Number (cells per section) of GFP-positive cells in the ipsilateral VL and CL. Scale bars, 1 mm (left) and 100 μm (right). All other conventions are as in Figure 1.

Following the vector injections into the SMA of macaques, we analyzed the density of GFP-positive cortical neurons in the contralateral SMA and the ipsilateral ventral premotor cortex (Figure 3a). Surprisingly, the mean density values in these cortical areas were quite low across the vectors (Figure 3c). Even when the NeuRet (FuG-E) but not the NeuRet (P440E) vector was injected at a higher titer (4.0 × 10^10^ gc/mL in comparison with 8.5 × 10^9^ gc/mL), the density of GFP-positive neurons was only slightly increased. In our analysis of thalamic neuron labeling, we found that only the NeuRet (FuG-E) vector injected at its higher titer yielded the appearance of a large number of GFP-positive cells in the VL and CL (Figure 3b,d). Overall, only limited numbers of cortical and thalamic neurons could be labeled retrogradely with all types of the vectors, except for the higher-titer NeuRet (FuG-E) vector.

### 3.5. Inflammatory/Immune Responses of HiRet and NeuRet Vectors

**Figure 4:**
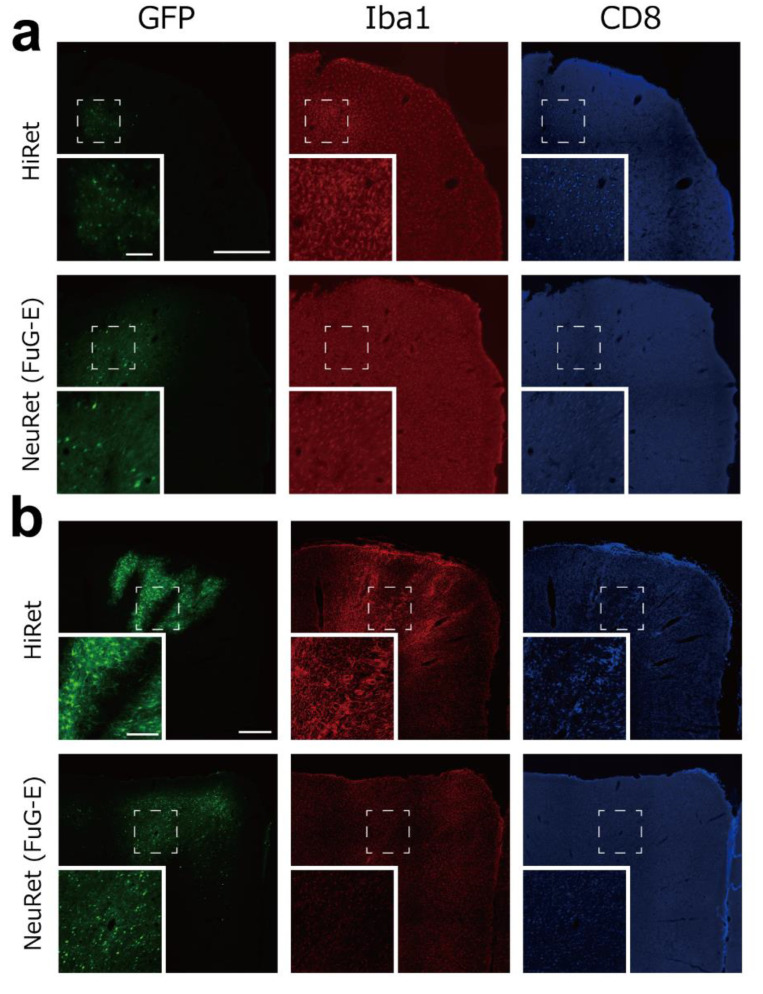

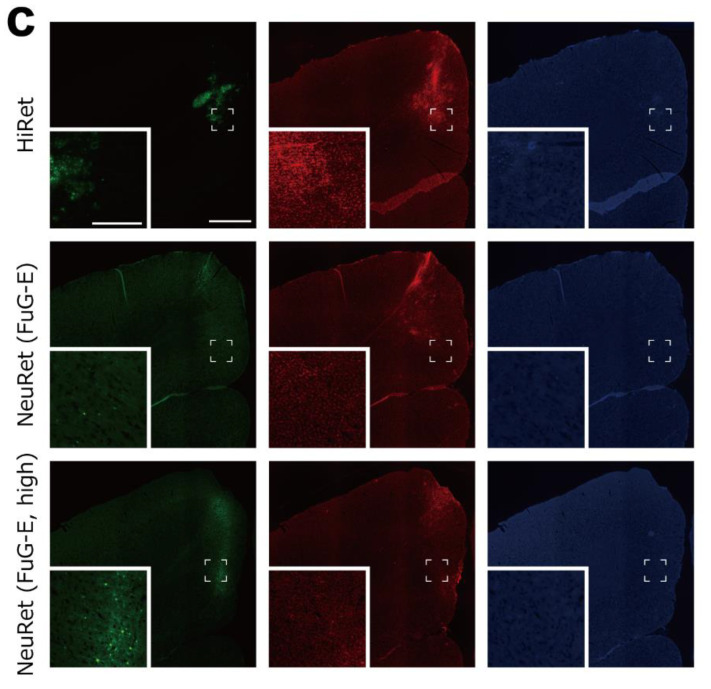
Inflammatory/immune responses of HiRet and NeuRet vectors. (**a**) GFP (green) and double immunofluorescence staining for Iba1 (red) and CD8 (blue) at the injection sites of the HiRet (upper) and NeuRet (FuG-E; lower) vectors in rats. Insets: higher-power magnifications of the square areas. Scale bars: 500 μm and 100 μm for insets. (**b**) Data obtained for marmosets. Scale bars: 500 μm and 200 μm for insets. (**c**) Data obtained for macaques. HiRet vector (top), NeuRet vector (middle), and higher-titer NeuRet vector (bottom). Scale bars: 2 mm and 0.2 mm for insets.

Subsequently, we explored inflammatory/immune responses of the HiRet and NeuRet (FuG-E) vectors at their injection sites in the rat, marmoset, and macaque brains. Double immunofluorescence histochemistry was performed to examine the occurrence of cells immunostained for Iba1 as a microglial marker or CD8 as a cytotoxic T cell marker around a GFP-positive cell population. With respect to the HiRet vector, infiltration of Iba1- and CD8-positive cells, indicative of inflammatory/immune responses, was observed in both the marmoset and the macaque brains, whereas no infiltration of Iba1- or CD8-positive cells was detected in the rat brain (Figure 4a–c). In remarkable contrast, the NeuRet vectors did not induce explicit signs of inflammatory/immune responses in either the marmoset or the macaque brain (Figure 4a–c).

### 3.6. Neuron Specificity of HiRet and NeuRet Vectors

**Figure 5:**
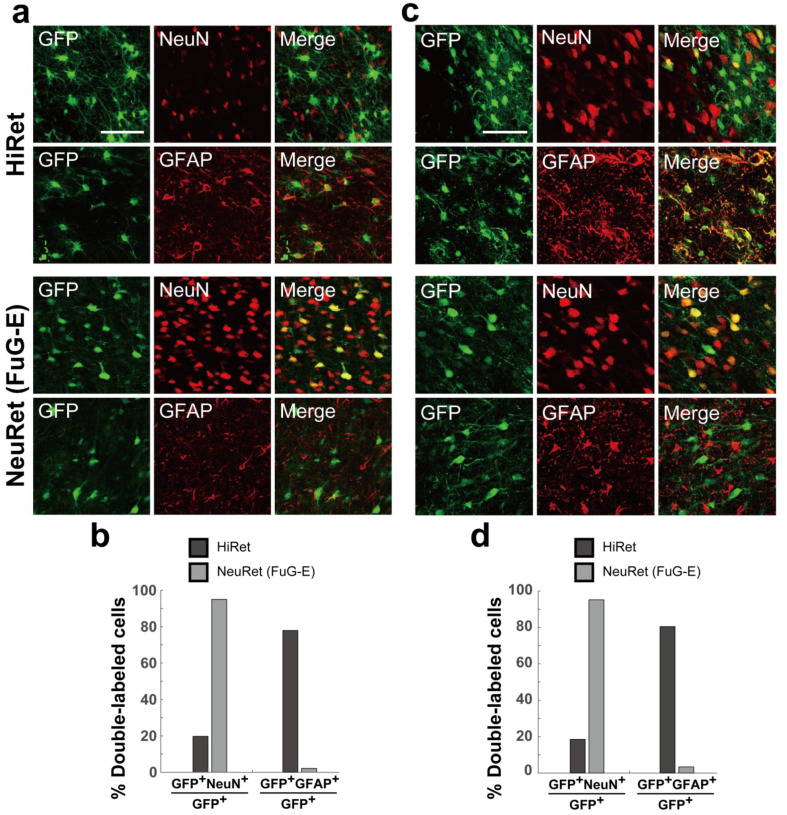
Neuron specificity of HiRet and NeuRet vectors. (**a**) Double immunofluorescence histochemistry for GFP and NeuN or GFP and GFAP at the injection sites of the HiRet (upper) and NeuRet (FuG-E; lower) vectors in marmosets. Scale bar, 50 μm. (**b**) Ratios of double-labeled cells (GFP^+^NeuN^+^, GFP^+^GFAP^+^) to the total GFP-positive cells (GFP^+^). (**c**,**d**) Data obtained for macaques. Scale bar, 50 μm.

Finally, we verified the neuron specificity of retrogradely transduced cells at the injection sites of the HiRet and NeuRet (FuG-E) vectors in the marmoset and macaque brains, since no marked inflammatory events occurred in the rat brain. Double immunofluorescence histochemistry was carried out to investigate the colocalization of NeuN as a neuronal marker or GFAP as an astroglial marker in GFP-positive cortical cells. The ratio of double-labeled cells to the total GFP-positive cells was calculated. In the case of the HiRet vector injection into the marmoset brain, a considerably large population (about 80%) of GFP-positive cells expressed GFAP, whereas only a small population (about 20%) of GFP-positive cells expressed NeuN. By contrast, the NeuRet (FuG-E) vector injection yielded NeuN expression in the vast majority (more than 95%) of GFP-positive cells (Figure 5a,b). Similar results were obtained after the vector injections into the macaque brain. In the case of the HiRet or NeuRet (FuG-E) vector injection, a large population of GFP-positive cells expressed GFAP or NeuN, respectively (Figure 5c,d).

## 4. Discussion

In our prior work [14], we determined that the NeuRet (FuG-E) vector is superior to the HiRet (FuG-B2) vector in terms of retrograde transgene efficiency, neuron specificity, and inflammatory events. It was clearly demonstrated in this work using the striatal input projections as a test system that the NeuRet vector is more meritorious than the HiRet vector for retrograde gene transfer into the primate brain as well as into the rodent brain, thereby indicating the validity of the NeuRet vector in long-term electrophysiological/behavioral experiments on primates. This prompted us to verify the usefulness of the NeuRet vector in another test system, the cortical input projections. Contrary to this supposition, the present results revealed that the NeuRet vector fails to display a high capacity of retrograde transgene expression in the macaque brain, especially for gene delivery into the intracortical connections.

Our histological analysis shows that the NeuRet vector generally exhibits a higher efficiency of retrograde gene transfer than the HiRet vector, except that the extent of retrograde transgene expression in the marmoset cortex is equivalent for both these vectors. The superiority of the NeuRet vector to the HiRet vector in the cortical input system is virtually consistent with the outcome seen in the striatal input system [14]. With respect to the intracortical connections, the NeuRet vector had much greater levels of retrograde transgene expression in the rat and marmoset brains than in the macaque brain, as described above. On the other hand, the efficiency of retrograde gene transfer into the thalamocortical projections via the NeuRet vector was similar and high across the three species, although the capacity of retrograde transgene expression in the macaque brain was somewhat low. For comparison, it should be noted here that, concerning the striatal input system we tested previously [14], the efficacy of retrograde gene transfer into the corticostriatal projections via the NeuRet vector is considerably low in the macaque brain, while the retrograde transgene efficacy toward the nigrostriatal and thalamostriatal projections is sufficiently high not only in the rat and marmoset brains, but also in the macaque brain.

The present data indicate that the NeuRet but not the HiRet vector permits highly neuron-specific gene transduction and, accordingly, causes no inflammatory/immune responses explicitly in the primate brain, which is in full agreement with the results obtained in our previous study [14]. It has been shown that AAV9 vector transduces genes into antigen-presenting cells expressing virus-derived proteins and provokes tissue damage in the rodent and primate brains, due to inflammation associated with microglial and lymphocytic infiltration [20,21]. In our histological confirmation, we observed infiltration of Iba1- and CD8-positive cells, representing inflammatory/immune responses, at the injection sites of the NeuRet vector in the marmoset and macaque brains. Intriguingly, no tissue damage like necrosis occurred in the rat brain, by the NeuRet vector nor by the HiRet vector, as previously reported in mice [11,22]. Thus, the NeuRet vector might be the primary choice for retrograde gene transfer experiments on primates.

In addition to the pseudotyped lentiviral vectors, other types of viral vectors, including recombinant glycoprotein-deleted RV vector, have been developed to allow retrograde gene transfer. Although these vectors were utilized for neural network tracing [23,24,25] and neuronal activity manipulation [26,27,28] and imaging [29,30,31], there were serious pitfalls concerning transgene efficacy and/or potential cytotoxicity, which made long-term experiments and clinical applications rather restricted. Different types of RV vectors, such as self-inactivated RV vector [32] and double-deletion-mutant RV vector [33], were also produced. However, retrograde transgene expression of these RV vectors was insufficient and transient, though their cytotoxicity became lower. Furthermore, a novel variant of adeno-associated virus vector, termed rAAV2-retro, has been introduced to yield transgene expression robustly in the retrograde direction [34]. This vector has been reported to possess stronger tropisms for specific cell types in mice than the double-deletion-mutant RV vector [33]. More recently, it has been demonstrated that rAAV2-retro can effectively be applied to neural networks related to the monkey cerebral cortex for their optogenetic/chemogenetic manipulation [35]. In this study, rAAV2-retro injected into the frontal cortex including the frontal eye field (FEF) could label neurons retrogradely in many cortical areas that receive input from the FEF, whereas almost no labeled neurons were observed in the thalamus. Thus, retrograde gene delivery into subcortical regions seems less efficient. It should also be mentioned here that a major disadvantage of AAV vectors is the limited packaging size of transgene (<5 kb) [36] as compared to a larger size for lentiviral vectors (<7.5 kb) [37].

In conjunction with our previous works [13,14], the present study elucidated that the NeuRet vector is more suitable than the HiRet vector for retrograde gene transfer by investigating the transgene expression patterns in the two major neural systems, i.e., the striatal and cortical input projections. Moreover, our results clarified that the efficiency of retrograde gene delivery for the NeuRet vector varies depending not only on the species, but also on the input projections. Of particular interest is that the modes of retrograde transgene expression, especially in the macaque brain, for the NeuRet vector and rAAV2-retro, may be arranged in a complementary fashion. With respect to the macaque brain, the NeuRet vector exhibits sufficient levels of retrograde transgene expression preferentially in the subcortical rather than the cortical structures, and vice versa for rAAV2-retro. This implies that close attention is required for selection of the vectors when experimental studies using retrograde gene transfer are designed for macaques. Though the differential neuronal tropisms of such vectors may be accounted for by postulating diverse membrane properties (e.g., receptor types) of infected neurons, their definitive causal mechanism remains unclear. In conclusion, the NeuRet vector pseudotyped with FuG-E might provide a valid tool for pathway-selective manipulation and monitoring of neuronal activity through reliable and stable retrograde gene transfer. This vector system could contribute to clarifying higher-order brain functions in primates and further serve to generate primate models of neurological/psychiatric disorders.

## Data Availability

The data supporting the findings of this study are available from the corresponding author upon request.
